# Commentary: Association between the *miR-146a* rs2910164 polymorphism and childhood acute lymphoblastic leukemia susceptibility in an Asian population

**DOI:** 10.3389/fgene.2023.1134659

**Published:** 2023-03-20

**Authors:** Abbas Navabi

**Affiliations:** ^1^ Department of Medical Biotechnology, School of Medical Sciences, Kermanshah University of Medical Sciences, Kermanshah, Iran; ^2^ Student Research Committee, School of Medical Sciences, Kermanshah University of Medical Sciences, Kermanshah, Iran

**Keywords:** ALL, miR-146a, rs2910164, Asian population, meta-analysis

## 1 Introduction

I read with great interest the valuable article titled “Association between the *miR-146a* Rs2910164 Polymorphism and Childhood Acute Lymphoblastic Leukemia Susceptibility in an Asian Population” published in the October 2020 edition of the journal (Zou et al., 2020). The authors included six studies based on their inclusion criteria. Their main finding indicated that the CC genotype significantly increased the risk of childhood acute lymphoblastic leukemia (ALL) in the additive model (CC vs. GG: OR = 1.598; 95% CI: 1.003–2.545; *p* = 0.049). Also, the dominant model, recessive model, and allele model indicated a trend of increasing risk for childhood ALL. However, there are some issues in the data extraction and meta-analysis that affect the results and must be noticed. Here, I aim to comment on the issues and provide accurate results through conducting a new meta-analysis. First, in Devanandan’s study, the genotyping method has been incorrectly recorded (Jemimah Devanandan et al., 2019). Second, in Xue’s study and Pei’s study, the frequency of the GG genotype has been defined as the frequency of the CC genotype and the frequency of the G allele has been defined as the frequency of the C allele, and *vice versa* (Xue et al., 2019; Pei et al., 2020). This has caused mistakes in statistical analysis and result interpretation. Third, in Pei’s study, all the participants were Taiwanese, not Taiwanese and Chinese (Pei et al., 2020). Therefore, in order to correct the findings of the meta-analysis by Zou et al. (2020), I used STATA 17.0 and CMA 3.0 software applications to conduct a meta-analysis based on the information reported in the original studies.

## 2 Results of my current meta-analysis

**FIGURE 1:**
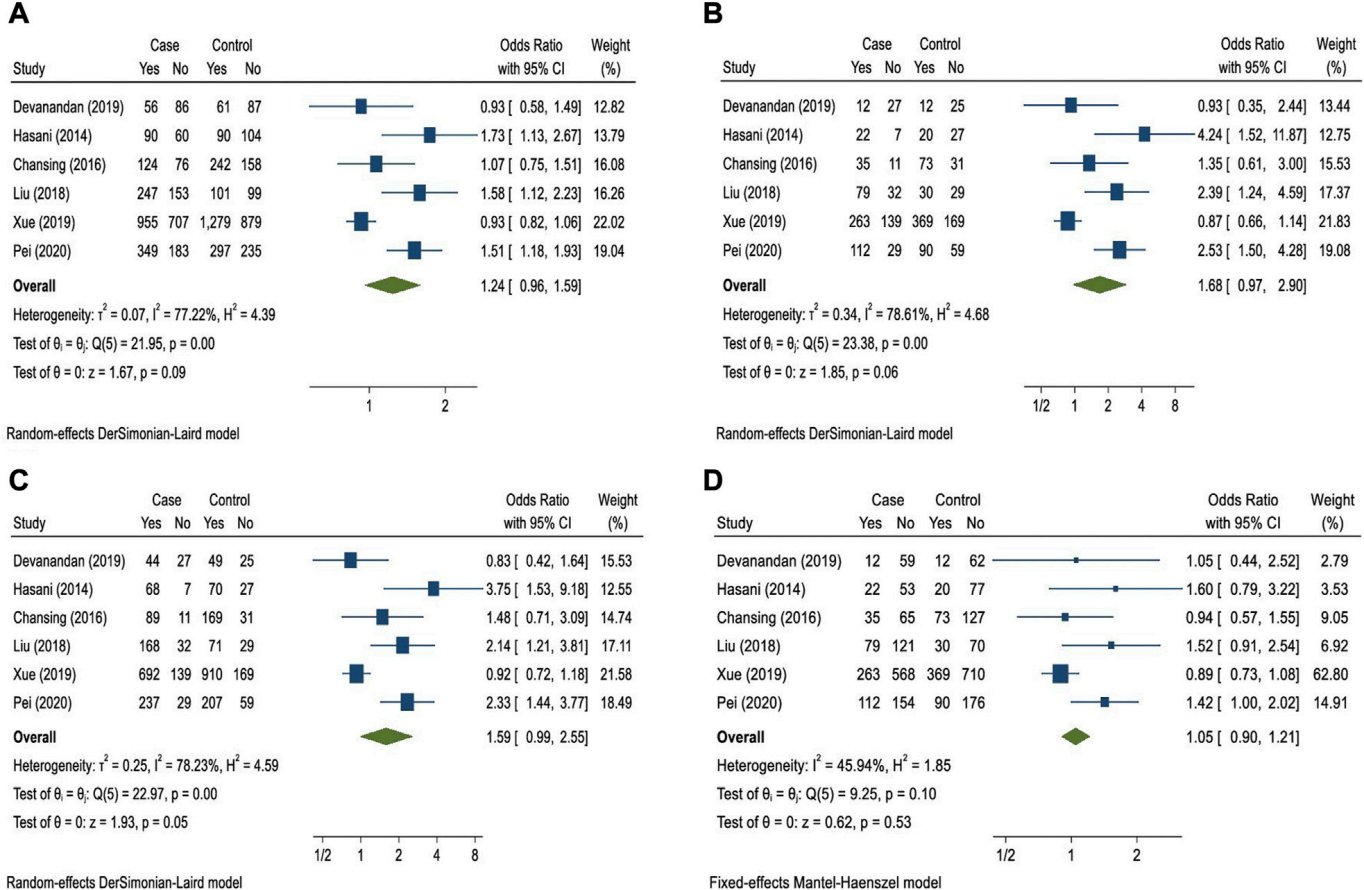
Forest plots for the allele **(A)**, additive **(B)**, dominant **(C)**, and recessive **(D)** models of the relationship between the miR-146a rs2910164 polymorphism and the risk of childhood acute lymphoblastic leukemia.

I present the correct characteristics of the included studies in Table 1 (Hasani et al., 2014; Chansing et al., 2016; Liu et al., 2018; Jemimah Devanandan et al., 2019; Xue et al., 2019; Pei et al., 2020). Based on the heterogeneity results of the meta-analysis of the association between rs2910164 and childhood ALL, except for the recessive model, the random effects model was used for meta-analysis. I obtained a pooled OR of 1.24 (95% CI: 0.96–1.59; *p* = 0.09) for the C allele in the allele model, 1.59 (95% CI: 0.99–2.55; *p* = 0.05) for the CC + CG genotype in the dominant model, 1.05 (95% CI: 0.90–1.21; *p* = 0.53) for the CC genotype in the recessive model, and 1.68 (95% CI: 0.97–2.90; *p* = 0.06) for the CC genotype in the additive model (Figure 1 and Supplementary Table S1). In all models, there was no significant association between the rs2910164 polymorphism and childhood ALL risk. Based on sensitivity analysis, removing the studies one by one from the included list showed that when Xue’s study is removed, the overall effect size of the different models changes significantly. Also, when Devanandan’s study is removed, the overall effect size of the dominant model changes significantly (Supplementary Figure S1 and Supplementary Table S2). According to the results of the funnel plot, Begg’s test, and Egger’s test, no publication bias was observed (Supplementary Figure S2 and Supplementary Table S3).

**TABLE 1:** Characteristics of six studies included in the present meta-analysis.

SNP	First author-year	Country	Continent	Genotyping method	Sample size		Genotype and allele distribution									
							Case					Control				
					Case	Control	GG	CG	CC	G	C	GG	CG	CC	G	C
rs2910164	Devanandan-2019	India	Asian	TaqMan	71	74	27	32	12	86	56	25	37	12	87	61
	Hasani-2014	Iran	Asian	T-ARMS-PCR	75	97	7	46	22	60	90	27	50	20	104	90
	Xue-2019	China	Asian	SNaPshot	831	1,079	139	429	263	707	955	169	541	369	879	1,279
	Chansing-2016	Thailand	Asian	PCR-RFLP	100	200	11	54	35	76	124	31	96	73	158	242
	Pei-2020	Taiwan	Asian	PCR-RFLP	266	266	29	125	112	183	349	59	117	90	235	297
	Liu-2018	China	Asian	PCR-RFLP	200	100	32	89	79	153	247	29	41	30	99	101

## 3 Conclusion

I conducted a meta-analysis based on information derived from six studies included in the meta-analysis by Zou et al. (2020) to assess the efficacy of the miR-146a rs2910164 polymorphism on childhood ALL risk. According to analysis, there was no significant association between the rs2910164 polymorphism and childhood ALL in all models. Based on sensitivity analysis after removing Xue’s study, childhood ALL risk was significantly increased in allele (C vs. G), additive (CC vs. GG), dominant (CC + CG vs. GG), and recessive (CC vs. CG + GG) models. Also, after removing Devanandan’s study, childhood ALL risk was significantly increased in the dominant model (CC + CG vs. GG). The results of the funnel plots, Egger’s test, and Begg’s test suggested that there is no obvious publication bias.
