# Supplementary material for: Commentary: Association between the miR-146a rs2910164 polymorphism and childhood acute lymphoblastic leukemia susceptibility in an Asian population
Source: Front Genet. 2023 Mar 20;14:1134659. doi: 10.3389/fgene.2023.1134659 (PMC10067635; doi:10.3389/fgene.2023.1134659)
Supplement: Supplementary file 1 [file DataSheet1.ZIP › Supplementary Figure 1.docx]

(A) (B)


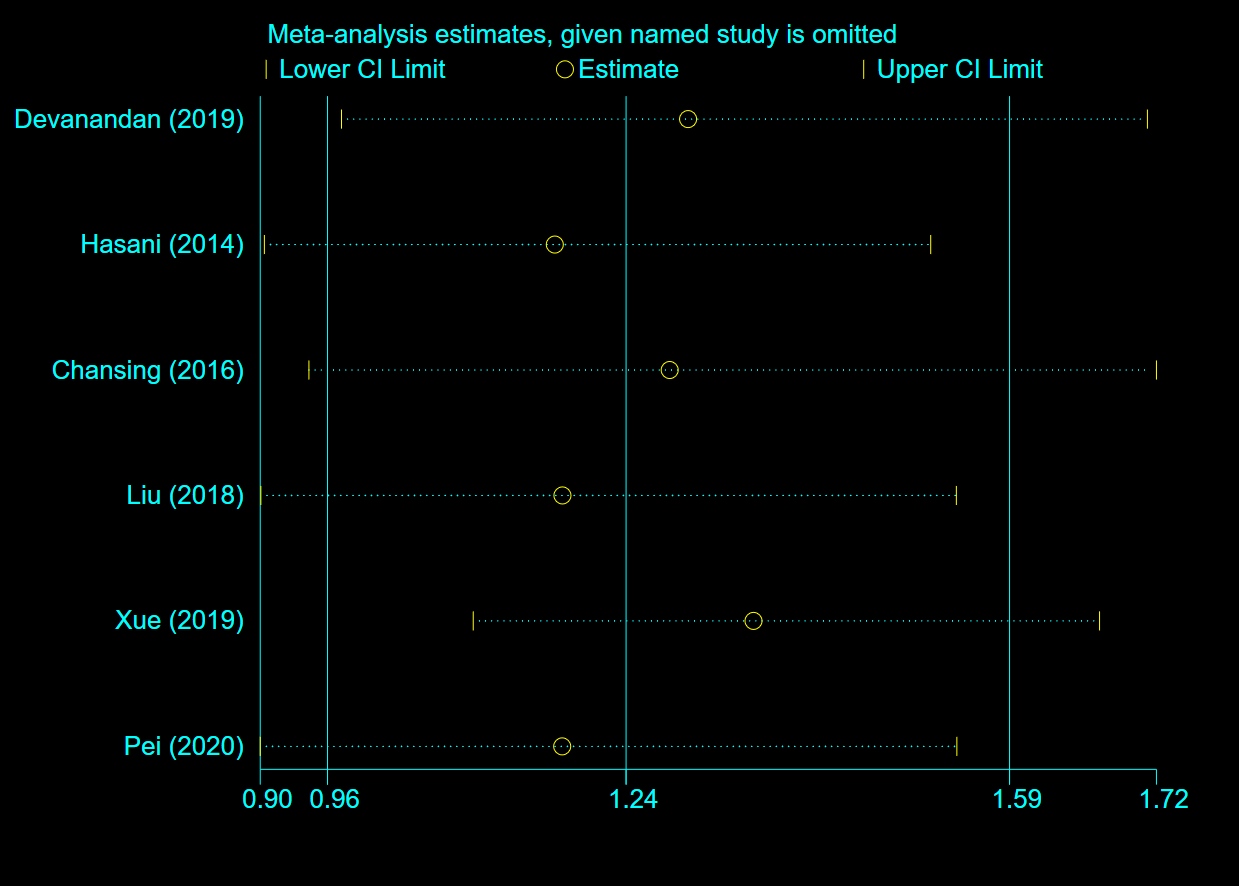

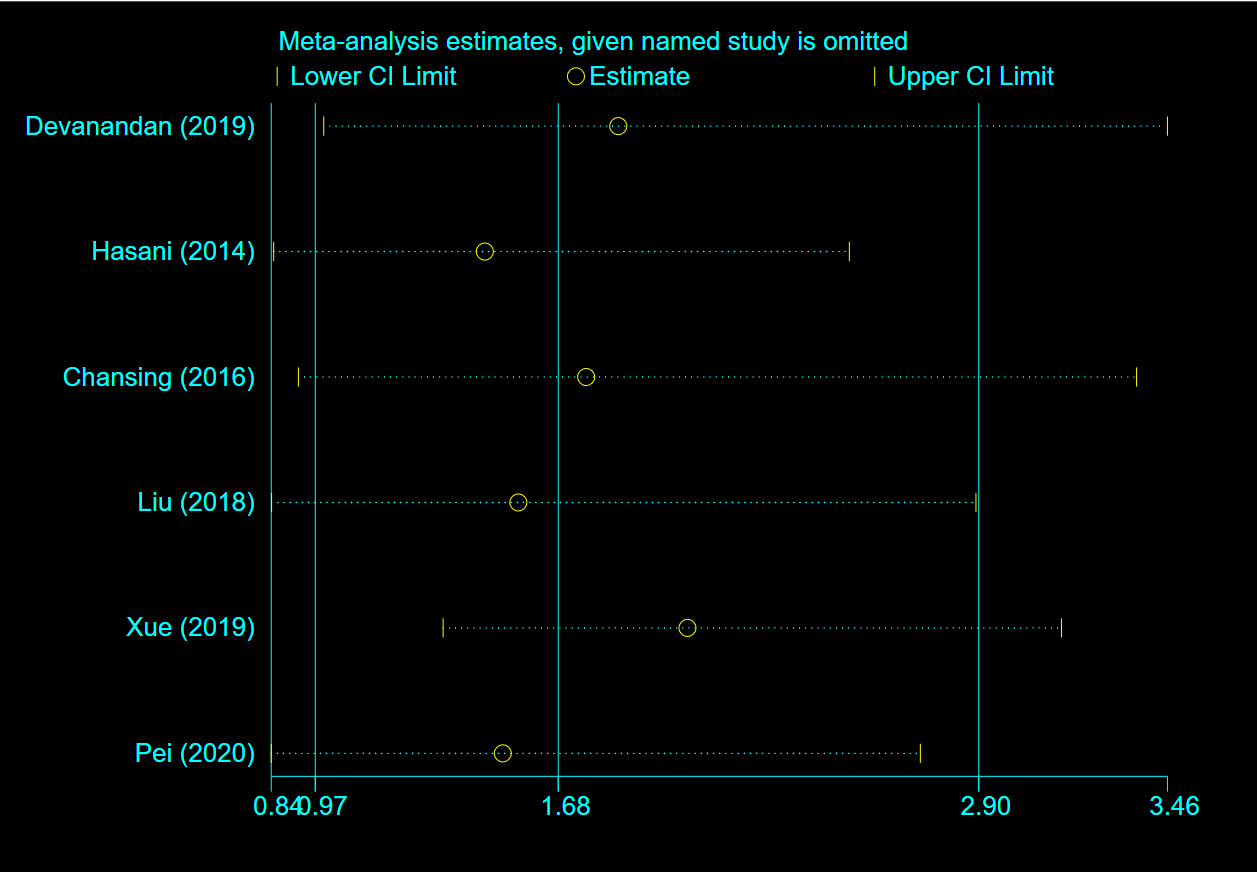


(C) (D)


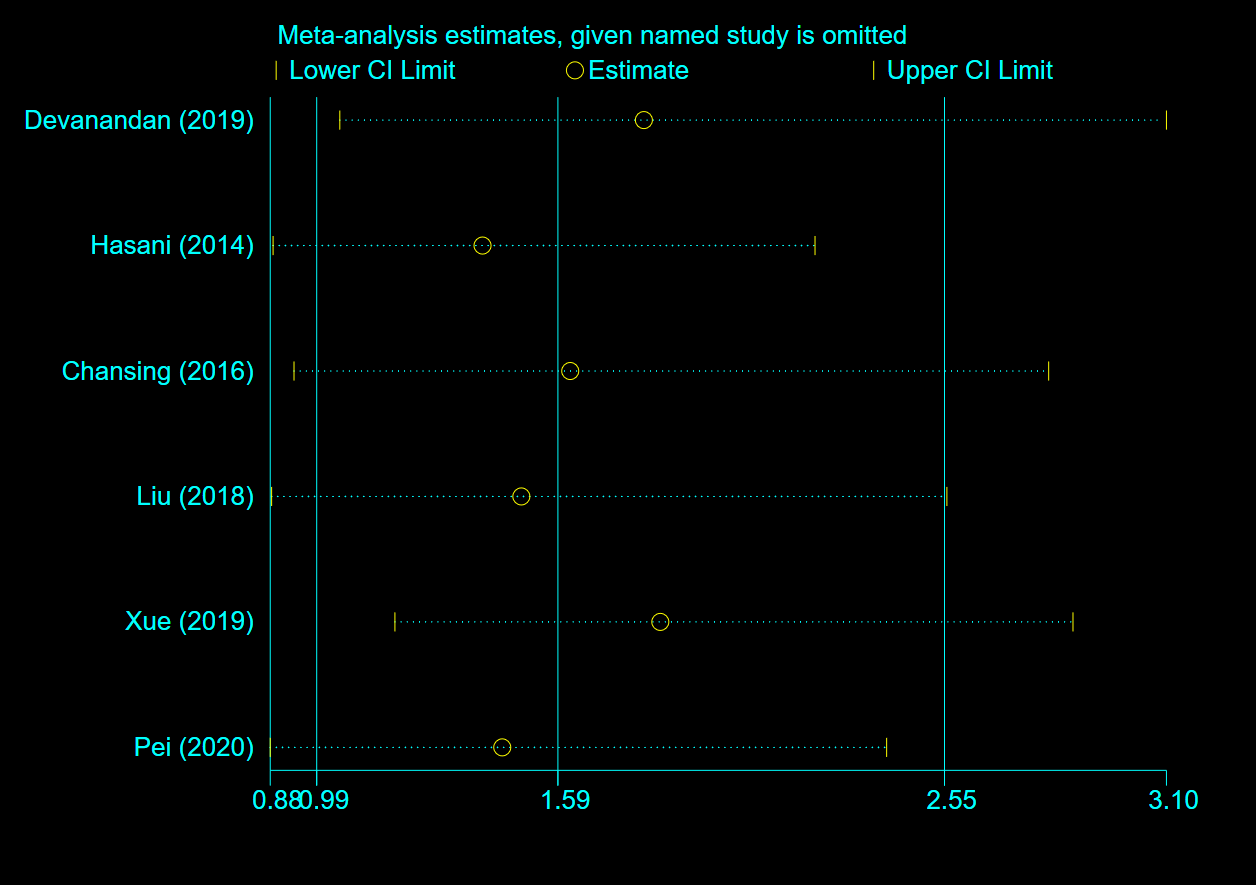

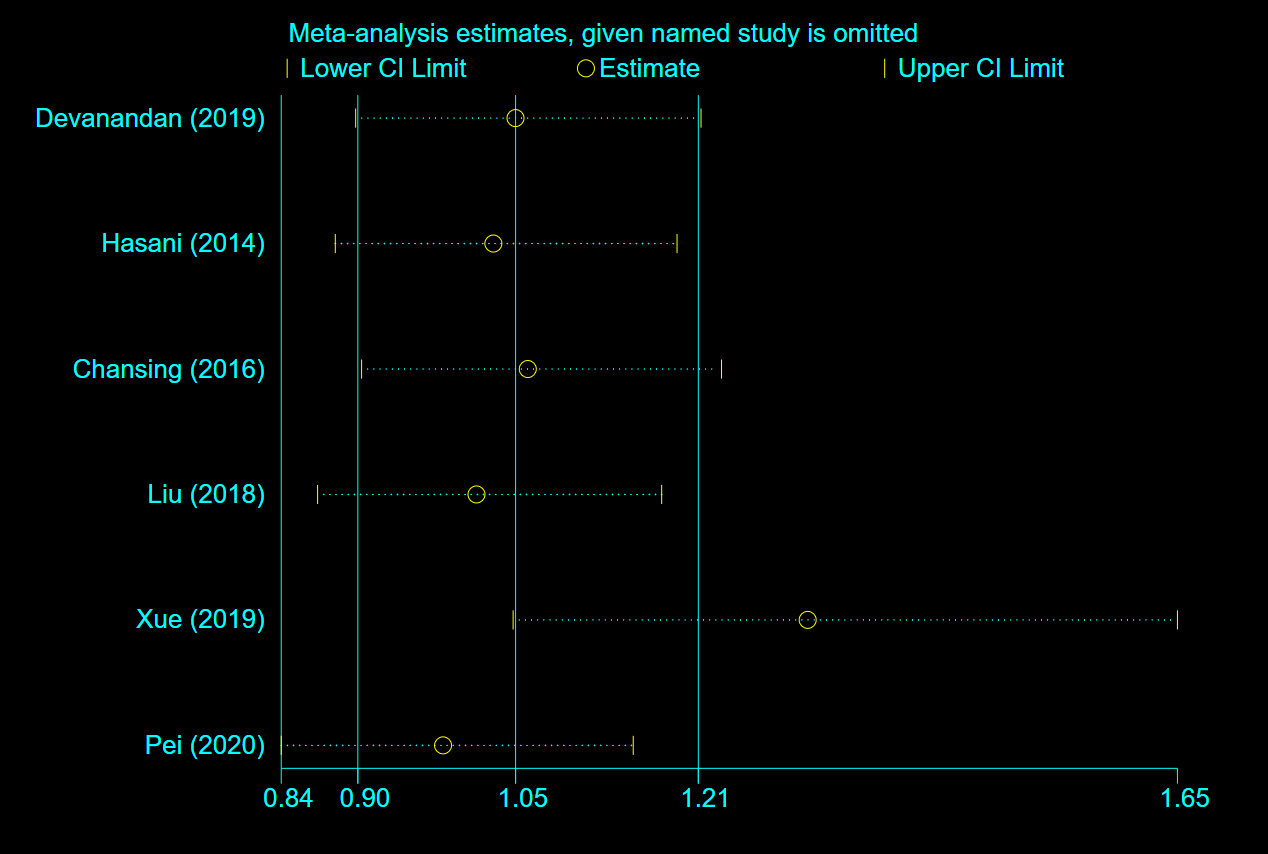


**Supplementary Figure 1.** Sensitivity analysis examining the association between the miR-146a rs2910164 polymorphism and risk of childhood acute lymphoblastic leukemia under the allele (A), additive (B), dominant (C), and recessive (D) models.
