# Supplementary material for: Commentary: Association between the miR-146a rs2910164 polymorphism and childhood acute lymphoblastic leukemia susceptibility in an Asian population
Source: Front Genet. 2023 Mar 20;14:1134659. doi: 10.3389/fgene.2023.1134659 (PMC10067635; doi:10.3389/fgene.2023.1134659)
Supplement: Supplementary file 1 [file DataSheet1.ZIP › Supplementary Figure 2.docx]

**A))**


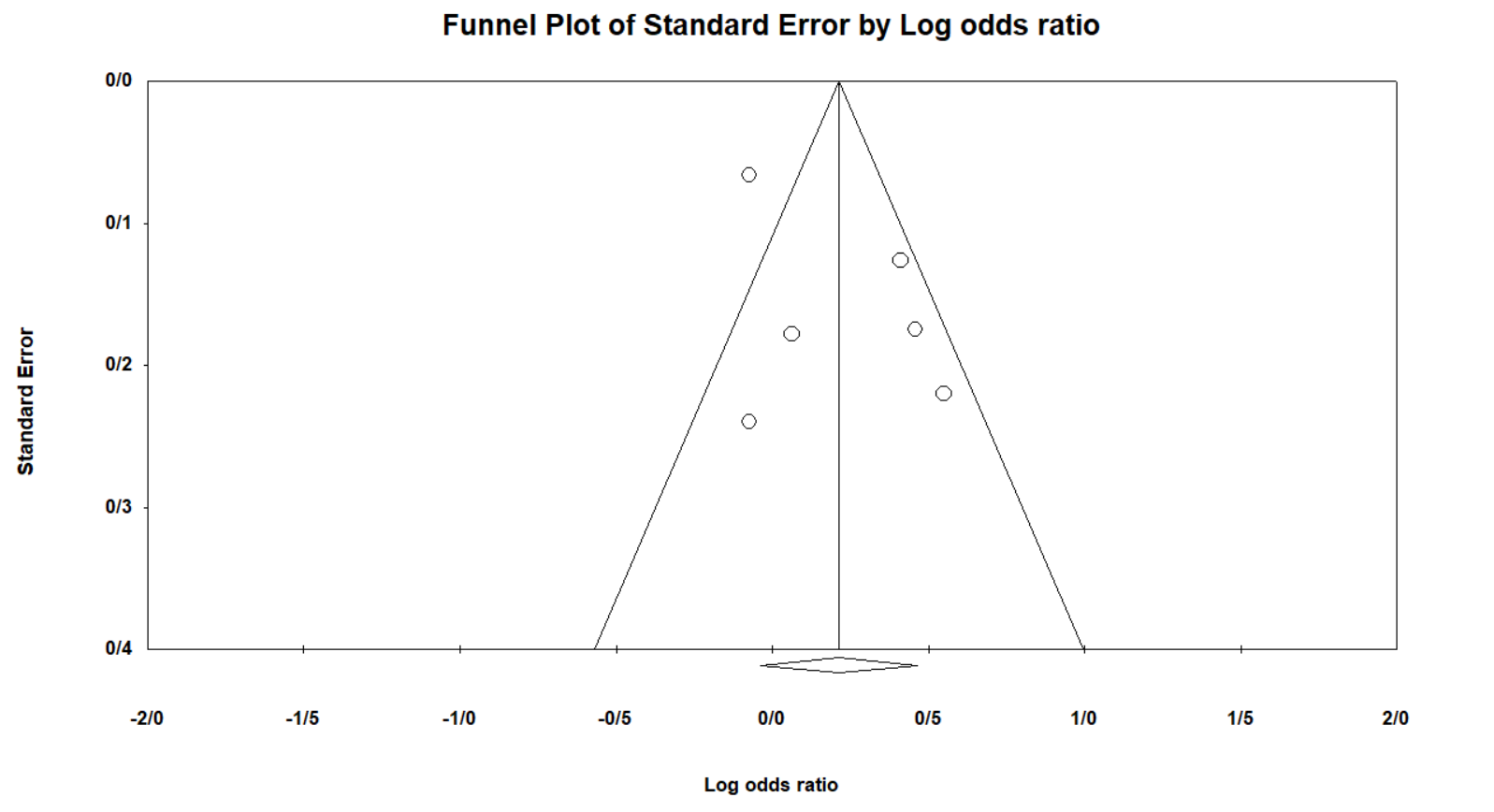


**(B)**

**
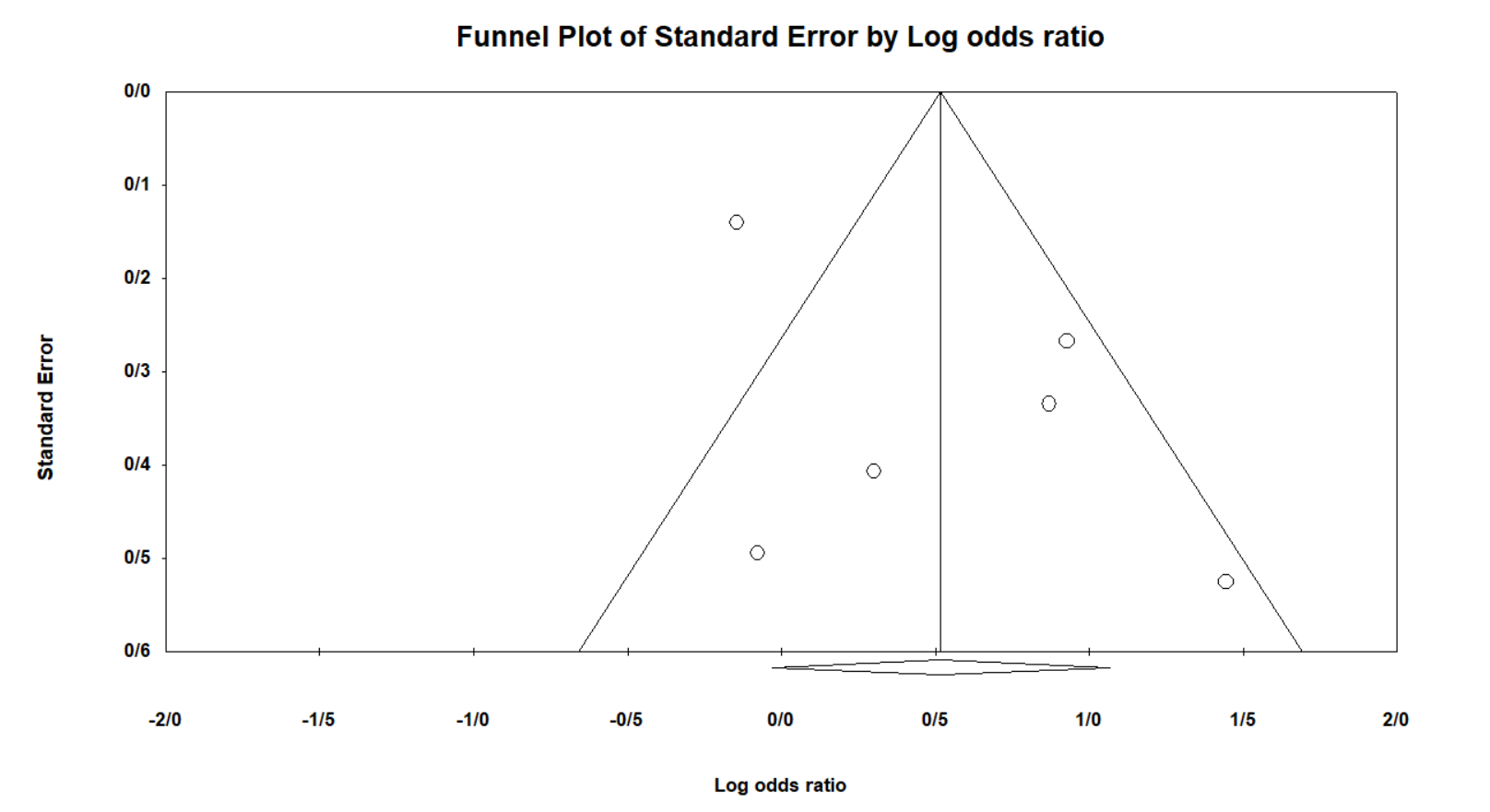
**

**(C)**

**
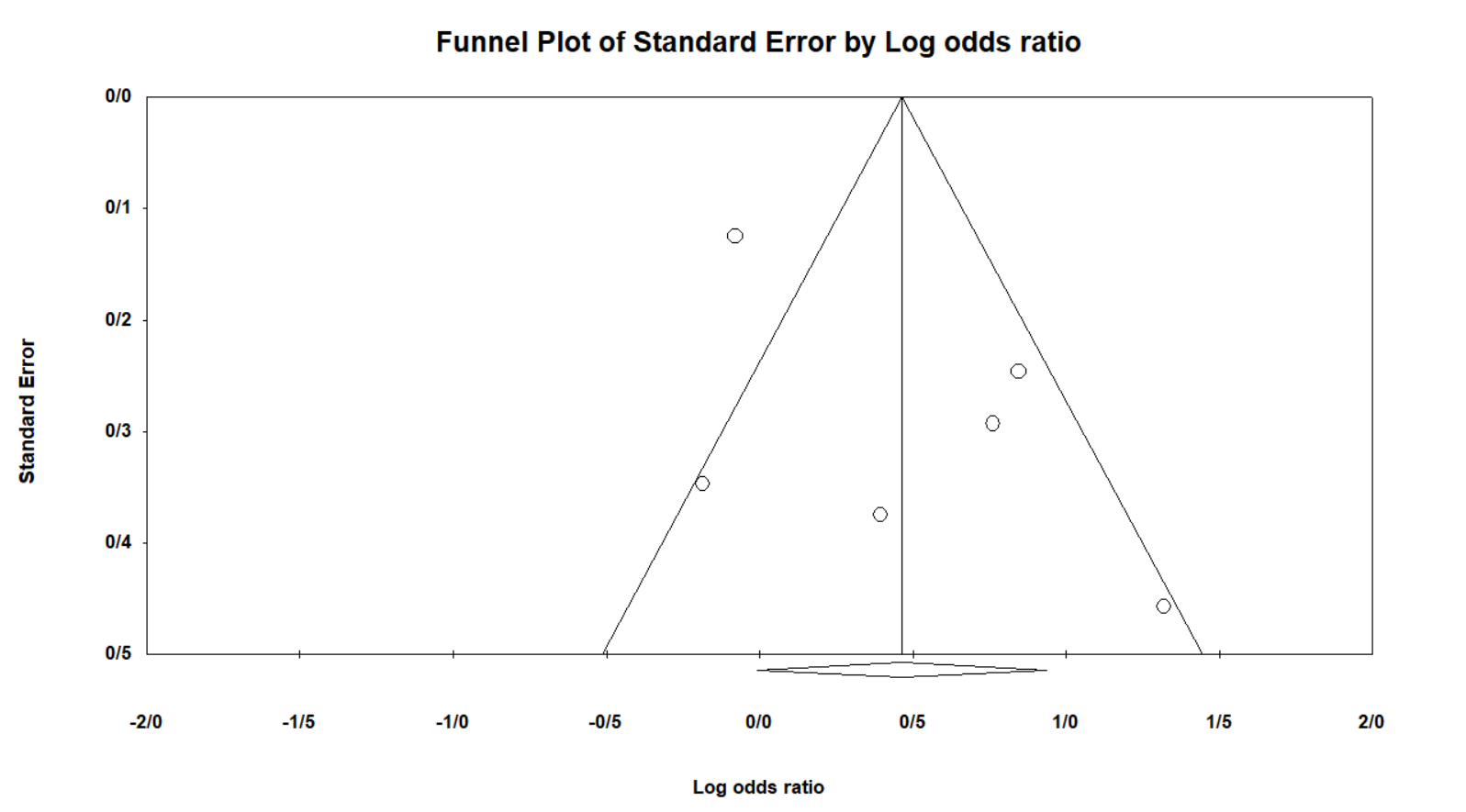
**

**(D)**


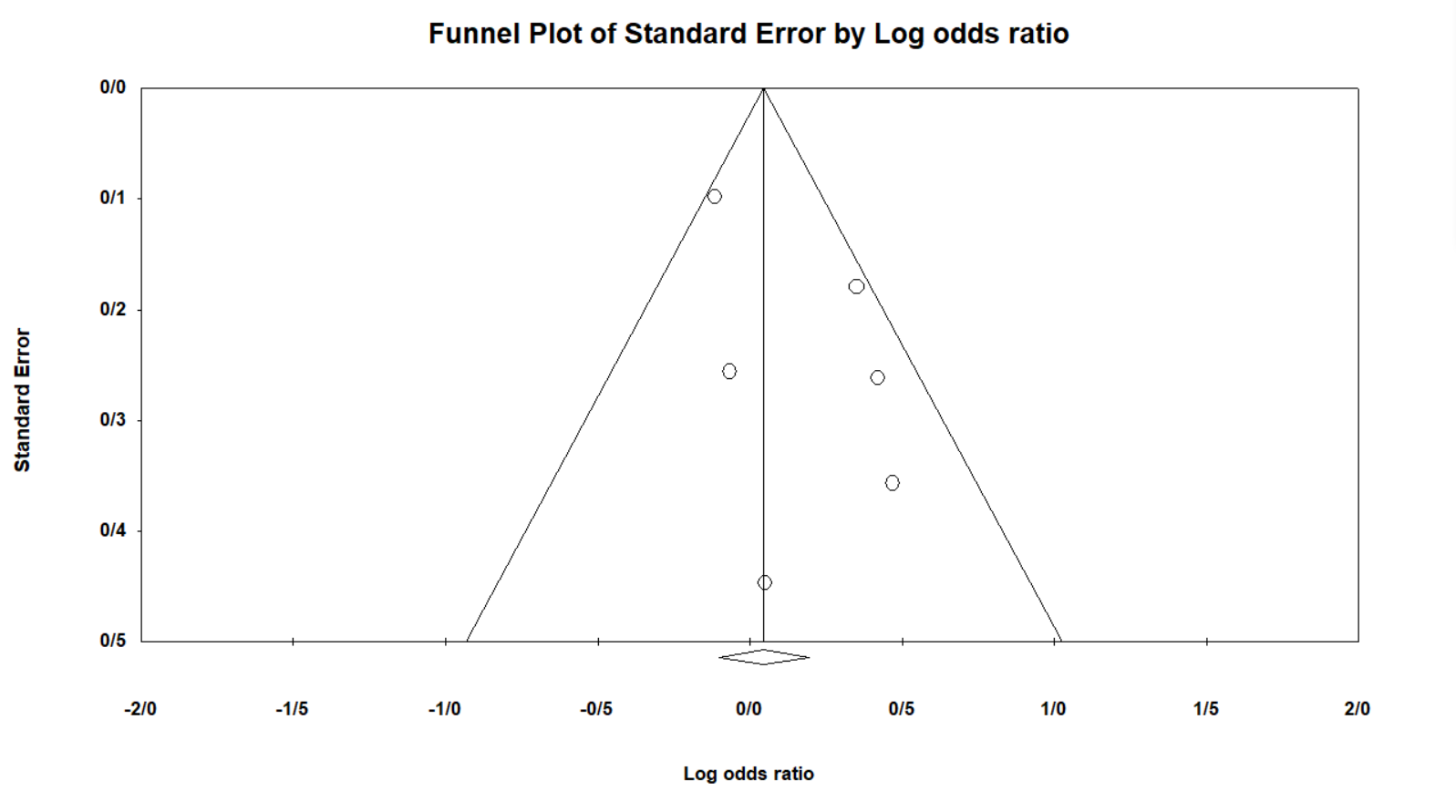


**Supplementary Figure 2.** Funnel plots for publication bias analysis of the association of miR-146a rs2910164 with the risk of childhood ALL under the allele (A), additive (B), dominant (C), and recessive (D) models.
