# Supplementary material for: Commentary: Association between the miR-146a rs2910164 polymorphism and childhood acute lymphoblastic leukemia susceptibility in an Asian population
Source: Front Genet. 2023 Mar 20;14:1134659. doi: 10.3389/fgene.2023.1134659 (PMC10067635; doi:10.3389/fgene.2023.1134659)
Supplement: Supplementary file 1 [file DataSheet1.ZIP › Supplementary Table 1.docx]

**Supplementary Table 1.** Meta-analysis of the association between the miR-146a (rs2910164) polymorphism and childhood acute lymphoblastic leukemia.

| Effect model | I^2^ | Q | 95% Cl | P-value | OR | study (n) | Comparison type |
| --- | --- | --- | --- | --- | --- | --- | --- |
| Random | 77/22% | 21.95 | 0.96 – 1.59 | 0.09 | 1.24 | 6 | C versus. G |
| Random | 78.61% | 23.38 | 0.97 – 2.90 | 0.06 | 1.68 | 6 | CC versus. GG |
| Random | 78.23% | 22.97 | 0.99 – 2.55 | 0.05 | 1.59 | 6 | CC + CG versus. GG |
| Fixed | 45.94% | 9.25 | 0.90 – 1.21 | 0.53 | 1.05 | 6 | CC versus. CG + GG |
