# Supplementary material for: Commentary: Association between the miR-146a rs2910164 polymorphism and childhood acute lymphoblastic leukemia susceptibility in an Asian population
Source: Front Genet. 2023 Mar 20;14:1134659. doi: 10.3389/fgene.2023.1134659 (PMC10067635; doi:10.3389/fgene.2023.1134659)
Supplement: Supplementary file 1 [file DataSheet1.ZIP › Supplementary Table 2.docx]

**Supplementary Table 2.** Results of sensitivity analysis by removing one study at a time.

| Study omitted | C vs. G | P-value | CC vs. GG | P-value | CC + CG vs. GG | P-value | CC vs. CG + GG | P-value |
| --- | --- | --- | --- | --- | --- | --- | --- | --- |
| Effect model | Random | | Random | | Random | | Fixed | |
| Devanandan | 1.2942721 | 0.073 | 1.8533757 | 0.052 | 1.8060098 | 0.032 | 1.0473888 | 0.550 |
| Hasani | 1.1720961 | 0.227 | 1.4641033 | 0.171 | 1.4052491 | 0.149 | 1.0273567 | 0.735 |
| Chansing | 1.2774185 | 0.109 | 1.7596996 | 0.088 | 1.6230841 | 0.084 | 1.058503 | 0.476 |
| Liu | 1.1790761 | 0.227 | 1.5618205 | 0.157 | 1.5016854 | 0.135 | 1.0120794 | 0.879 |
| Xue | 1.3543236 | 0.005 | 2.0551803 | 0.001 | 1.8468081 | 0.006 | 1.3117388 | 0.020 |
| Pei | 1.1788493 | 0.228 | 1.5164114 | 0.166 | 1.4542779 | 0.146 | .98182887 | 0.813 |
