# Supplementary material for: Commentary: Association between the miR-146a rs2910164 polymorphism and childhood acute lymphoblastic leukemia susceptibility in an Asian population
Source: Front Genet. 2023 Mar 20;14:1134659. doi: 10.3389/fgene.2023.1134659 (PMC10067635; doi:10.3389/fgene.2023.1134659)
Supplement: Supplementary file 1 [file DataSheet1.ZIP › Supplementary Table 3.docx]

**Supplementary Table 3.** Results of Begg’s test and Egger’s test to evaluate publication bias.

| Effect model | Egger’s test | Begg’s test | Comparison type |
| --- | --- | --- | --- |
| Random | P=0.4988 | P=0.7071 | C versus. G |
| Random | P=0.3617 | P=1.0 | CC versus. GG |
| Random | P=0.2296 | P=0.7071 | CC + CG versus. GG |
| Fixed | P=0.0566 | P=1.0 | CC versus. CG + GG |
